# Cytologically non-malignant thyroid nodules with three or more high-risk genetic mutations have poor outcomes

**DOI:** 10.1530/EO-25-0054

**Published:** 2025-10-07

**Authors:** Alexander R Goldberg, Cheng Liu, Gary D Rothberger, Kepal N Patel, Rong Xia, Steven Hodak, Sumedha Chablani

**Affiliations:** ^1^ Holman Division of Endocrinology, Diabetes, & Metabolism, Department of Medicine, NYU Grossman School of Medicine, New York, New York, USA; ^2^ Department of Pathology, NYU Grossman School of Medicine, New York, New York, USA; ^3^ Division of Endocrine Surgery, Department of Surgery, NYU Grossman School of Medicine, New York, New York, USA

**Keywords:** thyroid cancer, thyroid genetics, cytology, RAS mutation, TERT mutation, EIF1AX mutation

## Abstract

**Background:**

While traditional risk stratification for thyroid cancer primarily focused on tumor pathology, molecular profiling is increasingly recognized for its clinical relevance. There are limited data on tumors with three or more mutations, especially when presenting with non-malignant cytology. This study aims to evaluate the clinical behavior of cytologically non-malignant thyroid nodules with three or more potent oncogenic mutations.

**Methods:**

Electronic medical records of patients at our institution with thyroid nodules who underwent molecular testing were reviewed to identify cases with cytologically benign or indeterminate thyroid nodules that harbored three or more oncogenic mutations. Clinical, cytological, and molecular data were analyzed to assess tumor behavior.

**Results:**

Four of six cases were histologically malignant at index surgery. One histologically benign case developed distant metastases 7 years later. Retrospective analysis of the two histologically benign cases and the one case with low-risk histology demonstrated significant intratumoral heterogeneity. The benign case that developed distant metastases was found to have an area of intratumoral heterogeneity whose genetic profile matched the metastases.

**Conclusions:**

Despite bland cytological and ultrasonographic features, these tumors exhibited aggressive behavior. The molecular profile of thyroid cancers should be considered when determining treatment, especially in cases with multiple high-risk mutations, as they may behave more aggressively than predicted by cytology or imaging alone.

## Introduction

Staging and risk stratification for thyroid cancer traditionally relied on imaging findings and tumor pathology. However, molecular genetic testing is increasingly recognized as another very informative means by which thyroid cancer prognosis and risk stratification may be determined (1). It has previously been demonstrated that when thyroid cancers due to classic oncogenic drivers such as *BRAF* V600E and *RAS* co-occur with a second mutation, the increased genetic complexity of such tumors correlates with more aggressive disease behavior (2, 3). However, there is limited information on the disease trajectory of cytologically benign or indeterminate tumors with genetically high-risk profiles containing three or more genetic alterations known to be potent oncogenic mutations. This case series reports the cytologic, histologic, and overall clinical outcomes for patients at our institution with cytologically non-malignant thyroid nodules and three or more potent oncogenic mutations on molecular testing.

## Materials and methods

Electronic medical records of adult patients at our institution were reviewed to identify thyroid nodules with mutations identified between 2017 and 2023. Thyroid nodules with three or more genetically high-risk mutations (defined as previously reported potent oncogenic mutations) and non-malignant cytology were included. Non-malignant cytology was defined as category II to IV of the Bethesda System for Reporting Thyroid Cytopathology (4). Patient characteristics, thyroid ultrasonography, fine needle aspiration (FNA), cytology, molecular testing, surgical pathology, and outcomes were assessed. All cases, except for cases 1 and 5, had molecular testing done on thyroid cytology before surgery. Case 1 had initial molecular testing performed on distant metastasis, with subsequent molecular testing on a prior thyroid FNA cytology specimen. Case 5 had post-operative molecular testing performed on surgical pathology. Molecular testing in all cases was done with ThyroSeq®, allowing us to report the allelic frequencies of single nucleotide variants, which in all cases were well above the 5% threshold used to determine mutation clonality (5). Three cases (1, 3, and 4) with benign or ATA low-risk pathology underwent retrospective histological analysis of the surgical pathology.

## Results

We identified six patients at our institution from 2017 to 2023 with non-malignant pre-operative cytology, at least three potent mutations, and a confirmed surgical diagnosis (Table 1). This represents a frequency of 0.2% of non-malignant cytology samples that were sent for genetic testing in that time frame. Five primary tumors were histologically malignant and one was benign, with sizes ranging from 1.7 to 7.2 cm. The average age at diagnosis was 63 years (range: 34–84 years), and five of the six cases were female (Table 1).

**Table 1:** Patient characteristics.

Case	Age	Means of detection	Ultrasound	Bethesda class	Mutations	Histological type	Size (cm)	AJCC staging	Treatment	Original ATA risk	Response to therapy	Duration of follow-up (months)
	Gender											
1	70 F	Known nodule	Solid: hypoechoic	2	HRAS Q61K 44%	Initial: adenoma	3.5	IVb	Initial HT	Benign	Recurrence to thyroid bed and lateral neck	101
					TERT C228T 56%	Metastasis: FVPTC			CT and sternal mass resection		Sternal metastasis	
					EIF1AX A113_splice 45%				RAI			
					DICER1 E1705K 68%							
2	34 F	Neck swelling	Solid: hypoechoic	4	NRAS Q61R 33%	EFVPTC with extensive angioinvasion	1.7	I	TT	High	No current evidence of biochemical or structural disease	56
					TERT C250T 12%				RAI 147.9 mCi			
					CNA		2.2					
					GEA	EFVPTC						
3	54 F	Known nodule	Solid: hypoechoic	3	KRAS Q61R 26%	FTC with capsular invasion, no angioinvasion	6.0	I	TT	Low	No current evidence of biochemical or structural disease	12
					TERT C228T 38%				RAI 103.5 mCi			
					EIF1AX A113_splice 27%							
4	71 F	Known nodule	Solid: isoechoic	3	HRAS Q61R 41%	FA	2.4	n/a	TT	Benign	n/a	12
					TERT C228T 47%							
					EIF1AX A113_splice 35%							
5	84 M	Neck swelling	Cystic with mural solid component	2	NRAS Q61K 47%	FTC with extensive angioinvasion	7.2	II	HT, CT	High	Structural incomplete response	8
					TERT C228T 39%							
					EIF1AX A113_splice 85%							
6	66 F	Incidental	Solid: hypoechoic	3	NRAS Q61K 20%	PDTC with extensive angioinvasion	6.1	II	HT	High	No current evidence of biochemical or structural disease	4
					TERT C228T 26%							
					PIK3CA E542V 21%							
					GEA							

Abbreviations: M, male; F, female; FVPTC, follicular variant of papillary thyroid carcinoma; EFVPTC, encapsulated follicular variant of papillary thyroid carcinoma; FTC, follicular thyroid carcinoma; FA, follicular adenoma; PDTC, poorly differentiated thyroid carcinoma; HT, hemithyroidectomy; TT, total thyroidectomy; CT, completion thyroidectomy; RAI, radioactive iodine; GEA, gene expression alterations; CNA, copy number alterations.

Two cases presented with new neck enlargement. Four had asymptomatic presentations, including three cases with longstanding thyroid nodules with previously benign cytology. One case was identified during surveillance for breast cancer. None of the nodules identified had high-risk ultrasonographic features. The primary genetic mutation in all cases was a *RAS* mutation. All cases had a *TERT* mutation, and four cases had an *EIF1AX* mutation. Additional alterations included *DICER1*, *PIK3CA*, and copy number alterations (CNA).

Two cases were cytologically benign, and four were indeterminate (Table 1). Surgical pathology demonstrated five histologically follicular thyroid tumors: two follicular thyroid carcinomas (FTC), two follicular variant of papillary thyroid carcinomas (FVPTC), and one benign follicular adenoma (FA). The final patient presented with poorly differentiated thyroid cancer (PDTC) with extensive angioinvasion.

Three patients were ATA high-risk based on initial pathology, and one patient developed distant metastases post-operatively.

**Figure 1:**
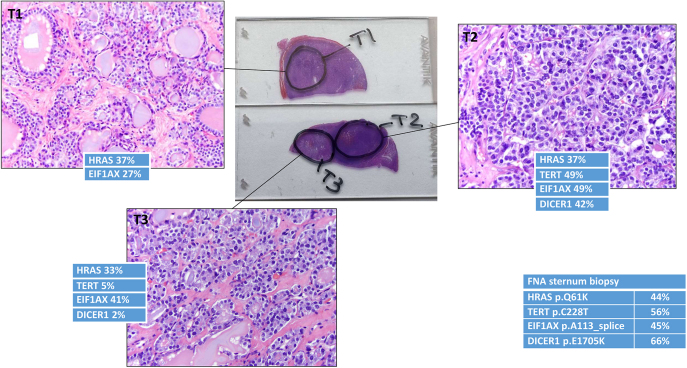
Case 1 histology and molecular testing: tumor subpopulations with relative hypocellularity (T1), hypercellularity (T2), and an intermediate area (T3). Each section was further analyzed by molecular testing and compared to the sternal metastasis molecular testing.

Case 1 had Bethesda II cytology and a 3.5 cm adenomatoid nodule treated with hemithyroidectomy but presented 7 years later with a 9 cm sternal metastasis and recurrence in the thyroid bed. Retrospective molecular testing on the 3.5 cm adenomatoid nodule demonstrated the presence of *RAS*, *TERT*, *EIF1AX*, and *DICER1* mutations, which was consistent with molecular findings in the metastasis, confirming that the metastasis had arisen from the original ‘benign’ adenoma (Fig. 1).

Further analysis of the benign and ATA low-risk cases (1, 3, 4) showed the presence of intratumoral heterogeneity with subpopulations demonstrating hypercellular microfollicular growth, hyperchromatic nuclei, and increased mitotic figures. For case 1, tumor subpopulations with relative hypocellularity, hypercellularity, and an intermediate area were further analyzed with molecular testing. The area with lowest cellularity had *HRAS* and *EIF1AX* mutations only. The intermediate area had additional *TERT* and *DICER1* mutations, but at low allelic frequency, whereas the hypercellular area showed higher allelic frequency of the *TERT* and *DICER1* mutations. The genetic profile of the late-presenting sternal metastasis matched that of the hypercellular area, suggesting that the metastasis had likely arisen from the subpopulation that expressed the oncogenes with the highest allelic frequency (Fig. 1).

Case 5 had Bethesda II cytology in a 7.2 cm cyst; however, surgical pathology demonstrated FTC with extensive angioinvasion in the solid mural component of the tumor. The molecular profile of this tumor demonstrating *RAS, TERT,* and *EIF1AX* mutations was confirmed post-operatively on the surgical specimen. Retrospective molecular testing of the pre-operative FNA cytology has not been possible.

Total thyroidectomy was the initial surgical management for three patients, two of whom received radioactive iodine (RAI). Lobectomy was performed in the remaining three patients. One patient had completion thyroidectomy and RAI following confirmation of FTC with extensive angioinvasion in the 7.2 cm cyst with benign pre-operative cytology. A second case was initially managed with lobectomy and underwent completion thyroidectomy 7 years later following presentation with sternal metastases and local recurrence. The patient with PDTC was managed with initial lobectomy and has declined completion thyroidectomy.

To date, four cases are recurrence-free; however, three have had 12 months or less of follow-up, including one who has not yet undergone repeat testing.

## Discussion

In this study, we explored the clinical and molecular characteristics of cytologically non-malignant thyroid tumors with genetically high-risk profiles. While thyroid carcinomas with multiple genetic alterations have been shown to have poor prognosis, there is limited data on cytologically non-malignant tumors with three or more mutations. In 2013, Nikiforova *et al.* reported that thyroid cancers with at least two mutations were at higher risk for distant metastasis and local recurrence (6). Song *et al.* have previously reported that *TERT* + *BRAF* cancers are larger with more extrathyroidal extension, and *TERT* + *RAS* cancers have higher recurrence rates (2). Research has also demonstrated a higher rate of distant metastases in cancers with *TERT* + *RAS* mutations (2, 7).

Our series of six cases is small, but our literature review found only seven cases of differentiated thyroid cancer and one case of FA with three or more mutations and non-malignant cytology (8, 9, 10, 11). These included three FTC, two FVPTC, one oncocytic carcinoma of the thyroid, and one ‘malignancy-low risk’ (either PTC, oncocytic carcinoma of the thyroid, or minimally invasive FTC). The most common mutations were *EIF1AX* (seven cases), *TERT* (six cases), and *RAS* (five cases). None of these cases had reported long-term follow-up.

Despite the high-risk molecular profile of the tumors in our study, they all presented with bland features. Except for a mixed solid/cystic nodule in case 5, the tumors were uniformly solid and iso- to hypoechoic with no high-risk ultrasonographic features. Similarly, cytologic features were also bland: two were benign, three were Bethesda III, and only one case was Bethesda IV.

These lower-grade features on presentation led to more conservative initial management, with hemithyroidectomy followed only later by completion thyroidectomy in two cases and recommended, but declined, thyroidectomy by the patient in a third case. For example, case 5 presented with a 7.2 cm mostly cystic nodule that was Bethesda II on cytology and was treated with lobectomy. However, based on surgical pathology, completion thyroidectomy was performed, and RAI was recommended as well. Similarly, case 1 was initially treated with lobectomy for a 3.5 cm nodule that was benign on cytology but required completion thyroidectomy, sternal mass resection, and RAI after a distant metastasis developed.

The discordance between the bland presentations and the high-risk genetic profile likely resulted from the fact that *RAS* was the primary driver mutation in all tumors. *RAS* mutations typically lead to tumors that maintain histologically follicular architecture and lack the typical nuclear characteristics of PTCs. As such, they most commonly have Bethesda III–IV cytology and lower-grade ultrasonographic features (1). Despite these bland ultrasonographic, cytologic, and histologic features, these tumors have a significantly increased risk of aggressive behavior due to the high-risk genetic alterations, further emphasizing the important role molecular genetic testing can play in tumor prognosis.

While three of the cases (2, 3, and 6) currently have no evidence of structural or biochemical recurrence on follow-up, two of these cases have had 12 or fewer months of follow-up. Two of these three cases were ATA high risk based on the presence of extensive angioinvasion in case 2 and PDTC in case 6, which conveys a significant lifetime risk of recurrence despite the current negative follow-up. In addition, case 3 was treated with RAI post-operatively due to the high-risk genetic profile despite being ATA low risk for recurrence, which may affect outcomes. While the current lack of disease is reassuring, these cases remain at high risk due to their genetic profile and will need close monitoring.

Two of the tumors with high-risk molecular profiles in our series were benign at initial histologic diagnosis (cases 1 and 4). Case 1 presented 7 years later with a sternal metastasis. Although case 4, which had benign follicular histology on final histology, continues to demonstrate no evidence of recurrence at 12 months post-operatively, the high-risk genetic profile nonetheless suggests a potential for future malignant behavior. This is consistent with the literature that has previously reported cases of metastases from histologically benign adenomas (12, 13, 14, 15, 16). We believe that if molecular testing had been available, a high-risk genetic profile would have been identified in these cases, explaining the eventual metastasis from these otherwise benign-appearing tumors.

Cases in our series were not histologically homogeneous and were noted to have subpopulations of hypercellularity with atypical follicular cells and high mitotic activity within the resected tumor. Case 1 had additional molecular testing of these subpopulations, which showed the area with hypercellularity and increased mitotic figures had the most high-risk genomic profile, with penetrance of the driver mutations at the highest allelic frequency. The similarity between the genomic landscapes of the sternal metastasis and the original tumor confirmed that this subpopulation was the metastatic origin and also suggested that the areas of intratumoral heterogeneity are responsible for malignant transformation (17, 18). These cases emphasize the important role molecular genetic testing can play in identifying the potential for malignancy and aggressive biologic behavior, even before it is reflected in tumor histology.

The lack of *BRAF*-driven tumors in this series is likely related to the fact that such cases typically present with higher-grade (Bethesda V or VI) FNA cytology, which typically does not undergo molecular genetic testing (1, 4). Since this series focused on cases with non-malignant cytology, *BRAF*-driven tumors with multiple mutations would not be expected. Although not captured here, *BRAF*-driven tumors with three or more mutations would be expected to have poorer prognosis, consistent with the previously reported literature on tumors with *BRAF* mutations co-occurring with another mutation (2, 7, 19).

In addition to *RAS*, *TERT* was the most common secondary driver mutation in this series and was present in all evaluated tumors. Multiple studies have previously shown *TERT* is associated with worse outcomes when occurring with *RAS*, even serving as an independent prognostic risk factor for disease-specific death among ATA high-risk cases (2, 20).

*EIF1AX* was the third most common mutation noted. While not highly oncogenic in isolation, there is evidence that it increases the risk of malignancy and aggressiveness when co-occurring with other mutations (8, 9, 10). *EIF1AX* has also been shown to augment the effects of *RAS* mutations through the mutual stabilization of c-MYC and mTOR activation (21).

This series has several limitations. The series is small, which reflects the rarity of these tumors. However, despite its limited size, it comprises the largest series of thyroid nodules with non-malignant cytology and three or more mutations reported to date. The average follow-up time for the cases in this series was relatively short: four of the six patients have less than 1 year of follow-up post-operatively, which prevents us from knowing whether any cases will also develop late recurrence or metastasis. *BRAF*-driven tumors are not captured in this series. Finally, the pre-operative molecular testing was available in four of the six cases and may have influenced treatment decisions and therefore impacted observed outcomes: case 3 was treated with RAI despite being ATA low-risk, while case 4 underwent total thyroidectomy rather than lobectomy based on the molecular findings in a 2.4 cm nodule that only had low-grade ultrasonographic features. In cases 1 and 5, the molecular tests were only available retrospectively. While they did not influence surgical management, these cases are important because they emphasize the significance of aggressive genetic signatures, even in benign cytology such as these two cases. Outcomes may be different if treatment decisions were based only on usual indices such as ultrasound appearance, less comprehensive molecular genetic testing, and histology.

While the literature has generally shown that DTC with multiple mutations is at higher risk for aggressive disease, data regarding cytologically non-malignant tumors with at least three genetically high-risk mutations is lacking. Given the clinically aggressive behavior of non-malignant tumors with three or more genetically high-risk mutations, our series suggests that when a high-risk genetic profile is present, primary surgical management with total thyroidectomy should be considered, even when imaging and cytology are not highly suspicious. Furthermore, because the tumors presented here are *RAS*-driven and are more likely to retain iodine avidity than *BRAF*-driven tumors, radioactive iodine may also be beneficial despite the high-grade histology. While the data presented here supports more aggressive management of thyroid nodules with non-malignant cytology and high-risk molecular profiles, more investigation is needed to better understand the long-term outcomes of these tumors.

## Declaration of interest

The authors declare that there is no conflict of interest that could be perceived as prejudicing the impartiality of the work reported.

## Funding

This work did not receive any specific grant from any funding agency in the public, commercial, or not-for-profit sector.

## Author contribution statement

SC and SH devised the project. SC, SH, ARG, GR, KP, and RX collected the data. SC, SH, and ARG performed the analysis, created the tables and figures, and wrote the article. All authors discussed the results and provided editorial review of the article.

## Ethics/informed consent

This is a retrospective review and analysis of patient data. As no direct patient contact or intervention occurred, informed consent was waived by the NYU Grossman School of Medicine (IRB i20-01314: Genomic Classifier Testing in Indeterminate Thyroid Nodules). All research involving human subjects complies with the Declaration of Helsinki.
